# From Local Tissue Repair to Fibrosis: Deciphering Gene Co-Expression Networks in Benign Pulmonary Nodules and Idiopathic Pulmonary Fibrosis Comorbidity via Bioinformatics and Machine Learning

**DOI:** 10.3390/ijms27083647

**Published:** 2026-04-19

**Authors:** Yaoyu Xie, Jingzhe Gao, Yifan Ren, Xiaoran Sun, Siju Lou, Guangli Yan, Ning Zhang, Hui Sun, Xijun Wang

**Affiliations:** Metabolomics Laboratory, Department of Pharmaceutical Analysis, Heilongjiang University of Chinese Medicine, National Chinmedomics Research Center, National TCM Key Laboratory of Serum Pharmacochemistry, State Key Laboratory of Integration and Innovation of Classic Formula and Modern Chinese Medicine, Heping Road 24, Harbin 150040, China

**Keywords:** benign pulmonary nodules, idiopathic pulmonary fibrosis, machine learning, bioinformatics, molecular docking

## Abstract

With increasing environmental pollution and a high incidence of respiratory infections, pulmonary nodules (PN) are being detected more frequently. Although most are benign, they are often accompanied by chronic inflammation and localized fibrosis, which may predispose patients to progression toward idiopathic pulmonary fibrosis (IPF). However, the biological relationship between benign pulmonary nodules (BPNs) and IPF remains poorly understood. Therefore, this study aims to investigate the shared molecular mechanisms and identify potential biomarkers linking BPN and IPF, with the goal of elucidating the pathogenic transition from BPN to IPF. In this study, microarray data from GEO datasets were systematically analyzed to explore shared molecular mechanisms, immune infiltration characteristics, and potential early intervention strategies linking BPN and IPF. Differential expression analysis, protein–protein interaction (PPI) networks, weighted gene co-expression network analysis (WGCNA), and integrative machine learning approaches identified *MME* and *ANKRD23* as key hub genes associated with the transition from BPN to IPF. Both genes demonstrated strong diagnostic performance, with Area Under the Curve (AUC) values exceeding 0.7, and were significantly correlated with immune cell infiltration, particularly effector memory CD8^+^ T cells. Functional enrichment and gene set enrichment analyses indicated that these genes were mainly involved in immune-related processes in BPN, while in IPF, *ANKRD23* was linked to cytoskeletal organization and genomic stability, and *MME* was enriched in profibrotic pathways such as TGF-β signaling. The diagnostic value of these biomarkers was further validated in a bleomycin-induced IPF mouse model using quantitative polymerase chain reaction (qPCR). In addition, drug–gene interaction prediction and molecular docking analyses highlighted several naturally derived compounds with favorable binding affinity and anti-inflammatory properties, among which folic acid, curcumin, and arbutin emerged as promising candidates for safe early intervention. Collectively, these findings identify *MME* and *ANKRD23* as potential biomarkers for early identification of BPN patients at risk of developing IPF and provide a theoretical basis for early diagnosis and targeted preventive strategies.

## 1. Introduction

With the widespread implementation of low-dose computed tomography (LDCT) screening, the detection rate of pulmonary nodules (PNs) in the general population has increased rapidly, emerging as a significant and increasingly recognized public health phenomenon. Epidemiological evidence from large-scale, population-based LDCT screening programs indicates that the overall prevalence of PN ranges from approximately 20% to 30%, of which more than 95% are ultimately confirmed to be benign through long-term radiological surveillance or histopathological evaluation, including granulomatous inflammation, intrapulmonary lymph nodes, and fibrotic scars [1,2]. Notably, the pathogenesis of benign pulmonary nodules (BPNs) is highly heterogeneous and multifactorial. Their development may be influenced by a wide range of factors, including environmental exposures such as inhaled particulate matter, prior infectious diseases—particularly tuberculosis, fungal infections, and bacterial pneumonia—genetic susceptibility, occupational exposures, and individual lifestyle characteristics [3,4]. At the pathophysiological level, these nodules are commonly associated with chronic low-grade inflammation, aberrant local tissue repair responses, and a predisposition toward interstitial fibrosis, reflecting the convergence of multiple biological pathways underlying benign nodule formation [5,6]. A CT-based study reported that, among BPN, 35% exhibited associated fibrotic lesions (FLs), defined as fibrotic tissue located adjacent to or within the nodule microenvironment [7].

IPF is a chronic, progressive interstitial lung disease marked by substantial clinical and pathological heterogeneity. The condition is characterized by irreversible fibrotic remodeling of the lung parenchyma, distortion of normal alveolar structures, and excessive accumulation of extracellular matrix components, ultimately resulting in progressive respiratory failure and an unfavorable prognosis [8]. During the early phase of disease development, patients commonly present with mild and nonspecific symptoms, such as persistent dry cough or exertional shortness of breath, which are frequently underestimated or mistaken for other, more prevalent respiratory disorders [9]. As a result, diagnosis is often delayed until substantial functional decline has occurred or extensive fibrotic abnormalities become evident on high-resolution imaging. By this stage, lung tissue damage is largely irreversible, and opportunities for early therapeutic intervention are significantly reduced [10,11]. At present, pharmacological management of IPF relies mainly on antifibrotic agents, including nintedanib and pirfenidone. These treatments have been shown to attenuate the rate of lung function deterioration but do not reverse established fibrosis, underscoring their role as disease-modifying rather than curative therapies [12,13]. In addition, treatment efficacy and tolerability vary widely among patients, and adverse effects frequently limit long-term adherence. With ongoing disease progression, individuals with IPF are at increased risk of developing serious comorbid conditions, such as pulmonary hypertension, right heart failure, and lung malignancies, all of which further complicate clinical management and contribute to increased mortality [14,15].

Bioinformatics enables the systematic dissection of complex biological processes and facilitates the identification of key molecular features underlying disease pathophysiology. At the molecular level, both BPN (specifically associated with pulmonary sarcoidosis) and IPF originate from aberrant programs of pulmonary epithelial cell repair and tissue remodeling, and share inflammatory signaling pathways centered on mediators such as tumor necrosis factor-α (TNF-α), interleukin-6 (IL-6), and transforming growth factor-β (TGF-β) [16,17]. The molecular convergence observed between BPN and IPF suggests that, under specific pathological or microenvironmental conditions, BPN may evolve toward more extensive fibrotic remodeling rather than remaining a static, localized lesion. Accordingly, integrating bioinformatics with machine learning and molecular docking approaches provides a powerful multidimensional strategy for systematically interrogating complex molecular interaction networks and for predicting biomarkers with potential clinical translatability. Such an integrative research framework is of significant scientific value for elucidating the latent connections between BPN and PF, enabling early identification of high-risk populations, timely clinical intervention, and ultimately improved patient outcomes.

## 2. Results

### 2.1. Integrated Transcriptomic Profiling Reveals 136 Genes Commonly Dysregulated and the Associated Enriched Pathways in BPN and IPF

**Figure 1:**
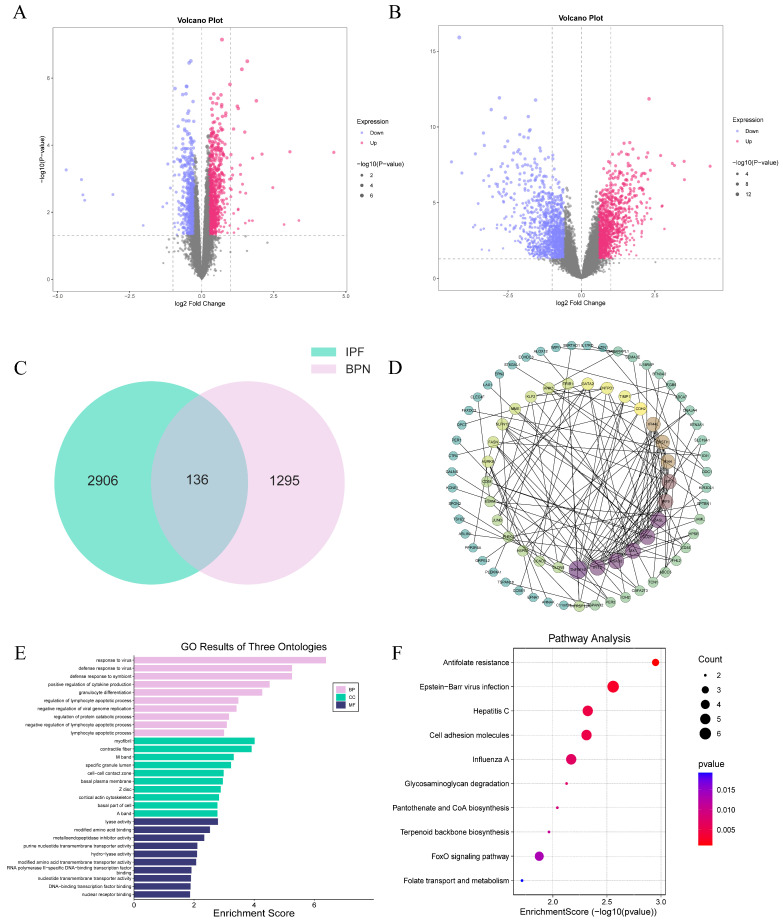
Screening of DEGs. (**A**): Volcano plot of DEGs in the BPN group; (**B**): Volcano plot of DEGs in the IPF group; (**C**): Venn diagram of differentially expressed genes between IPF and BPN; (**D**): PPI network of common differentially expressed genes in co-morbidities; (**E**): GO enrichment analysis of co-morbid common differential genes; (**F**): KEGG enrichment analysis of co-morbid common differential genes.

The microarray data for IPF (GSE10667) and BPN (GSE135304) were normalized to remove systematic variations between samples, thereby enhancing the suitability of the data for subsequent analyses (Figure S1A,B). After eliminating duplicate genes and excluding those with low expression, 19,749 genes with expression levels greater than zero were detected in IPF, among which 3042 were identified as differentially expressed genes (DEGs), including 1481 upregulated and 1561 downregulated genes. Similarly, in BPN, a total of 31,414 genes with expression levels exceeding zero were identified, of which 1431 were classified as DEGs, with 795 upregulated and 636 downregulated genes. Volcano plots were used to visualize the DEGs (Figure 1A,B). The intersection of the differential genes between the two diseases was then visualized using a Venn diagram, leading to the identification of 136 common genes for further investigation (Figure 1C). Further analysis of the intersection genes was conducted through protein–protein interaction (PPI) network analysis. Notably, genes such as *TNFSF10*, *RSAD2*, *OASL*, *MX1*, *IFIT3*, *CD27*, *IRF9*, *IFIT1*, *IFI44L*, *IFI44*, *EPSTI1*, *CDH2*, *TIMP1*, *GATA2*, *ENTPD1*, *TRIB1*, *SLFN13*, *MME*, *KLF2*, *FASN*, *AURKB*, and *ANK3* exhibited relatively high degrees of interaction (degree 5–15) (Figure 1D). These genes may serve as potential biomarkers and therapeutic targets. GO and KEGG enrichment analyses revealed that the co-DEGs in both IPF and BPN were primarily enriched in pathways related to viral infections (Epstein–Barr virus infection, Hepatitis C, and Influenza A), tissue fibrosis (myofibrils and contractile fibers), enzyme activity alterations (lyase activity, metalloendopeptidase inhibitor activity, purine nucleotide transmembrane transporter activity, hydro-lyase activity, and modified amino acid transmembrane transporter activity), cell adhesion molecules, and the FoxO signaling pathway (Figure 1E,F).

### 2.2. Construction of a Weighted Gene Co-Expression Network and Identification of Key Modules in BPN

**Figure 2:**
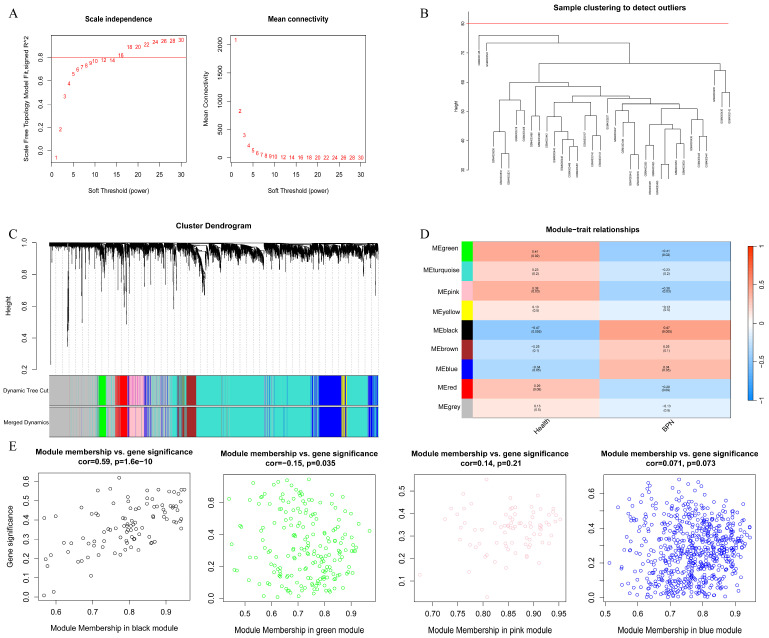
WGCNA of BPN. (**A**): Soft-thresholding power analysis based on the scale-free topology fit index and mean connectivity; (**B**): Hierarchical clustering dendrogram of samples showing no outliers; (**C**): Gene dendrogram and identification of co-expression modules using the dynamic tree-cut algorithm; (**D**): Module–trait correlation heatmap revealing associations between gene modules and the BPN phenotype; (**E**): Scatter plots of module eigengenes illustrating the significant correlations of the MEblack, MEgreen, MEpink, and MEblue modules with the BPN phenotype.

WGCNA was performed on the BPN dataset GSE135304. Based on the scale-free topology fit index and mean connectivity analysis, the optimal soft-thresholding power was determined to be 16, at which the scale-free topology index *R*^2^ = 0.80 (Figure 2A). Hierarchical clustering analysis demonstrated clear sample clustering without any detectable outliers (Figure 2B). Using the dynamic tree-cut algorithm, a total of 9 distinct gene co-expression modules were identified (Figure 2C).

Notably, the MEblack, MEgreen, MEpink and MEblue modules showed significant correlations with the BPN phenotype, each exhibiting an absolute correlation coefficient greater than 0.3 and statistical significance at *p* ≤ 0.05 (Figure 2D,E). Collectively, these four key modules contained 1014 genes, which were considered candidate module genes most closely associated with BPN-related phenotypic alterations.

### 2.3. Construction of a Weighted Gene Co-Expression Network and Identification of Key Modules in IPF

**Figure 3:**
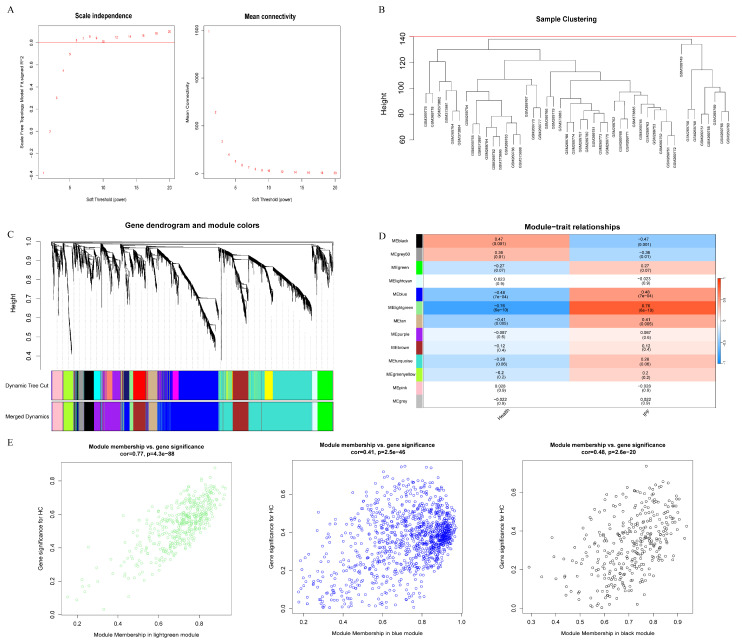
WGCNA of IPF. (**A**): Soft-thresholding power analysis based on the scale-free topology fit index and mean connectivity; (**B**): Hierarchical clustering dendrogram of samples showing no outliers; (**C**): Gene dendrogram and identification of co-expression modules using the dynamic tree-cut algorithm; (**D**): Module–trait correlation heatmap between gene modules and the IPF phenotype; (**E**): Scatter plots showing significant correlations of MElightgreen, MEblue, and MEblack modules with the IPF trait.

WGCNA was subsequently performed on the IPF dataset GSE10667 to elucidate gene co-expression architectures characteristic of IPF. Based on the scale-free topology fit index and mean connectivity analysis, the optimal soft-thresholding power was determined to be 6, at which the scale-free topology index *R*^2^ = 0.80 (Figure 3A). Hierarchical clustering analysis demonstrated clear sample clustering without any detectable outliers (Figure 3B). Using the dynamic tree-cut algorithm, a total of 13 distinct gene co-expression modules were identified (Figure 3C).

Notably, the MElightgreen, MEblue, and MEblack modules showed significant correlations with the IPF phenotype, each exhibiting an absolute correlation coefficient greater than 0.3 and statistical significance at *p* < 0.01 (Figure 3D,E). Collectively, these three key modules contained 1883 genes, which were considered candidate module genes most closely associated with IPF-related phenotypic alterations.

### 2.4. Identification of Core Overlapping Genes Shared Between BPN and IPF

**Figure 4:**
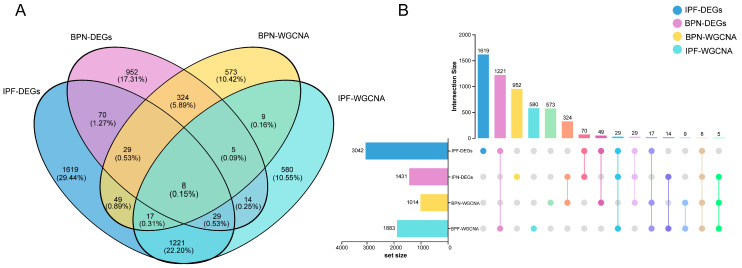
Identification and visualization of shared genes between IPF and BPN. (**A**): Interactive Venn diagram showing the overlap among IPF-DEGs, BPN-DEGs, and the key WGCNA modules in both diseases; (**B**): UpSet plot providing a clearer visualization of the pairwise and higher-order intersections among the four gene sets, enabling detailed comparison of two-way, three-way, and four-way overlaps.

The Venn diagram illustrates the overlap among IPF-DEGs, BPN-DEGs, and the key gene modules identified by WGCNA in IPF and BPN. The central intersection of the four gene sets comprises eight genes—*ANKRD23*, *IFIT1*, *MME*, *KCNE1L*, *AMICA1*, *ORM2*, *C13orf15*, and *OASL*. These genes are not only differentially expressed in both conditions but also consistently present within their respective co-expression modules. The existence of these common genes may reflect the progression of BPN to IPF, or indicate pathological mechanisms common to the two diseases (Figure 4A,B).

### 2.5. Machine Learning Identifies MME and ANKRD23 as Central Hub Genes in Both BPN and IPF

Using three machine learning algorithms, including Least Absolute Shrinkage and Selection Operator (LASSO), Random Forest (RF) and Support Vector Machine (SVM), we performed feature selection and key gene identification in both the BPN and IPF datasets.

**Figure 5:**
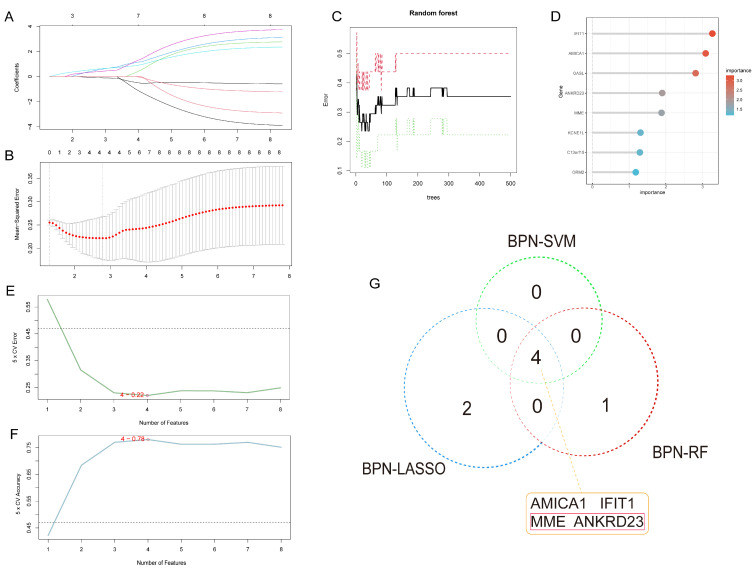
Identification of core biomarkers for BPN using three machine learning algorithms. (**A**): LASSO coefficient path plot: Different colored lines represent different features (genes included in the model). Each line shows the dynamic path of the regression coefficient for that feature as model complexity (the number of features/λ value) changes; (**B**): Cross-validation curve of the LASSO regression analysis: Two vertical dashed lines represent two key λ (penalty coefficient) values selected from cross-validation. The left dashed line corresponds to lambda.min—the λ value at which the cross-validated mean squared error (MSE) is minimized, giving the model with the smallest prediction error. The right dashed line corresponds to lambda.1se—the λ value within one standard error of the minimum error, yielding the most parsimonious model (fewest features). The red points represent the cross-validated MSE curve, showing how the average prediction error changes with model complexity (number of features/λ value). The lowest point of the curve corresponds to the optimal number of features; (**C**): Error curve of the RF algorithm: The black solid line represents the overall out-of-bag (OOB) error of the model, i.e., the average classification error rate across all samples, serving as the core metric for evaluating overall model performance. The red dashed line represents the OOB error for the control group, reflecting the model’s misclassification rate for that class. The green dashed line represents the OOB error for the disease group, reflecting the model’s misclassification rate for that class; (**D**): Ranking of important genes predicted by the RF algorithm; (**E**): Error rate of the SVM algorithm: The baseline error dashed line represents the error level of a random guessing model; (**F**): Accuracy of the SVM algorithm: The baseline accuracy dashed line represents the accuracy level corresponding to random guessing; (**G**): Core genes jointly predicted by the three machine learning algorithms.

In the BPN dataset, LASSO regression reduced the eight initial candidate genes to six key genes: *C13orf15*, *ANKRD23*, *ORM2*, *IFIT1*, *AMICA1*, and *MME* (Figure 5A,B). The RF algorithm subsequently identified the top five genes ranked by importance: *IFIT1*, *AMICA1*, *OASL*, *ANKRD23*, and *MME* (Figure 5C,D). SVM modeling demonstrated that inclusion of *IFIT1*, *AMICA1*, *MME*, and *ANKRD23* yielded the highest accuracy and lowest error rate (Figure 5E,F). By integrating the results from all three methods, *IFIT1*, *AMICA1*, *MME*, and *ANKRD23* were designated as potential biomarkers for BPN (Figure 5G).

**Figure 6:**
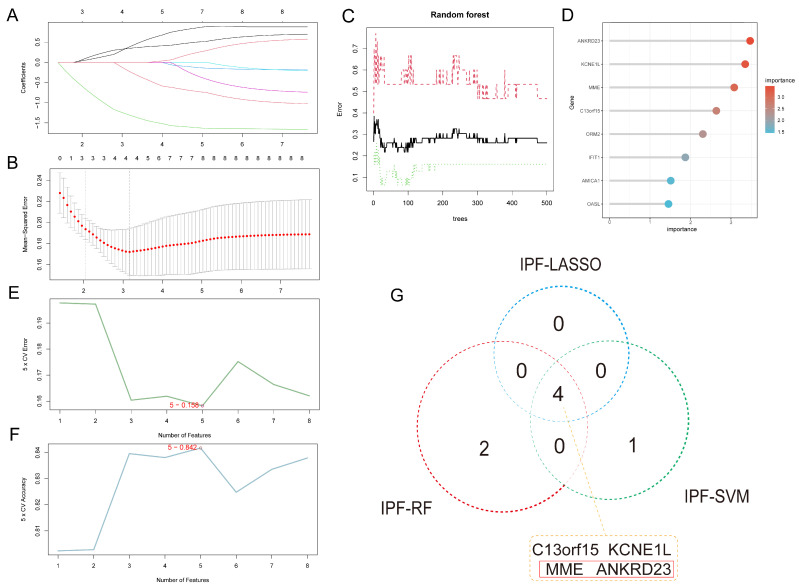
Identification of core biomarkers for IPF using three machine learning algorithms. (**A**): LASSO coefficient path plot; (**B**): Cross-validation curve of the LASSO regression analysis; (**C**): Error curve of the RF algorithm; (**D**): Ranking of important genes predicted by the RF algorithm; (**E**): Error rate of the SVM algorithm; (**F**): Accuracy of the SVM algorithm; (**G**). Core genes jointly predicted by the three machine learning algorithms.

In the IPF dataset, LASSO regression selected four key genes, namely *C13orf15*, *KCNE1L*, *ANKRD23*, and *MME*, from the initial pool of candidates (Figure 6A,B). RF analysis further identified the six most important genes: *ANKRD23*, *KCNE1L*, *MME*, *C13orf15*, *ORM2*, and *IFIT1* (Figure 6C,D). Results from the SVM model indicated that inclusion of five genes (*ANKRD23*, *C13orf15*, *KCNE1L*, *MME*, and *AMICA1*) achieved the best predictive performance, with the highest accuracy and lowest error rate (Figure 6E,F). After integrating the results from all three machine learning approaches, four overlapping genes, including *C13orf15*, *KCNE1L*, *MME*, and *ANKRD23*, were identified as potential biomarkers for IPF (Figure 6G).

Taken together, the machine learning analyses highlight *MME* and *ANKRD23* as the two most critical shared biomarkers in the comorbidity landscape of BPN and IPF.

### 2.6. Receiver Operating Characteristic (ROC) Curve Validation and Nomogram of the Core Genes

**Figure 7:**
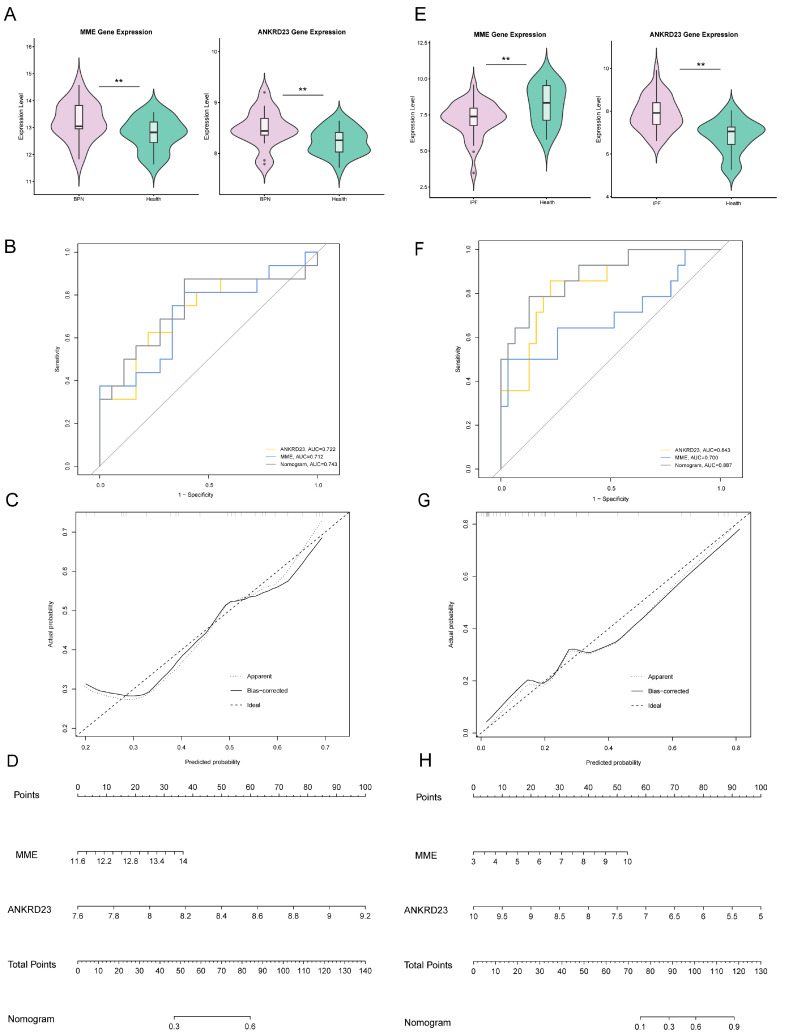
Assessment of the diagnostic and predictive value of *MME* and *ANKRD23* in BPN and IPF. (**A**): Violin plot of *MME* and *ANKRD23* expression in BPN patients and normal controls; (**B**): ROC curves of *MME* and *ANKRD23* for diagnosing BPN; (**C**): Calibration curve of the nomogram for BPN risk prediction; (**D**): Nomogram for predicting BPN risk based on *MME* and *ANKRD23*; (**E**): Violin plot of *MME* and *ANKRD23* expression in IPF patients and normal controls; (**F**): ROC curves of *MME* and *ANKRD23* for diagnosing IPF; (**G**): Calibration curve of the nomogram for IPF risk prediction; (**H**): Nomogram for predicting IPF risk based on *MME* and *ANKRD23*. ** *p* < 0.01.

Significant dysregulation of *MME* and *ANKRD23* was observed in patient cohorts compared to normal controls (Figure 7A,E). Specifically, BPN patients exhibited marked upregulation of both genes (*p* < 0.01), whereas in IPF patients, *MME* was significantly downregulated and *ANKRD23* was upregulated (*p* < 0.01). ROC analysis confirmed the strong diagnostic utility of these markers. For BPN, Area Under the Curve (AUC) values reached 0.712 for *MME* and 0.722 for *ANKRD23*. For IPF, the AUC was 0.700 for *MME* and 0.843 for *ANKRD23*, with all values exceeding the 0.7 threshold, indicative of substantial diagnostic power (Figure 7B,F). The calibration curves, validated by 1000 bootstrap resamples from both cohorts, demonstrated excellent agreement between predictions and observations (Figure 7C,G). This robust predictive performance was further reflected in the nomograms, where both genes served as significant contributors to the model’s predictive value for each disease (Figure 7D,H).

### 2.7. Analysis of Immune Cell Infiltration in the Microenvironment of BPN and IPF

**Figure 8:**
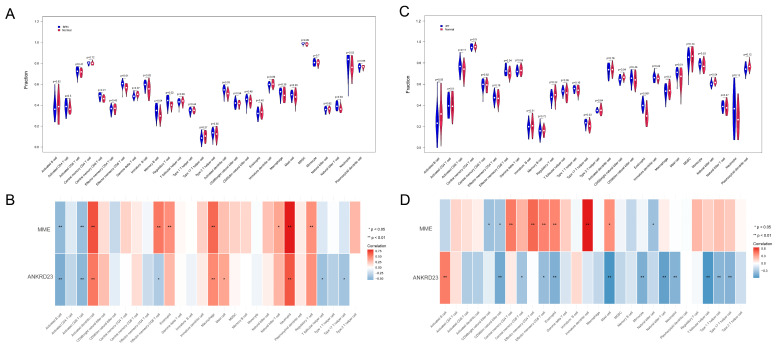
Immune infiltration landscape and core gene–immune cell associations in BPN and IPF. (**A**): Differentially infiltrated immune cell subsets in BPN patients, with eight of the 28 immune cell types showing significant alterations; (**B**): Correlation heatmap illustrating the association between core genes (*MME* and *ANKRD23*) and immune cell populations in BPN; (**C**): Differentially enriched immune cell subsets in IPF patients, with eight immune cell types exhibiting significant changes; (**D**): Correlation heatmap showing the relationship between *MME* and *ANKRD23* and immune cell infiltration in IPF.

To elucidate the functional involvement of immune cells in the pathogenesis and progression of BPN and IPF, we systematically evaluated immune infiltration patterns across the respective datasets. In BPN patients, eight out of the 28 immune cell subsets exhibited significant alterations (*p* < 0.05), including central memory CD8^+^ T cells, effector memory CD8^+^ T cells, immature B cells, memory B cells, regulatory T cells, activated dendritic cells, MDSCs, and natural killer T cells (Figure 8A).

Similarly, in the IPF cohort, eight immune cell populations were found to be differentially enriched (*p* < 0.05), specifically activated B cells, effector memory CD8^+^ T cells, T follicular helper cells, type 17 T helper cells, CD56bright natural killer cells, eosinophils, mast cells, and natural killer cells (Figure 8C).

Correlation heatmap analysis further revealed that *MME* and *ANKRD23* displayed a notable association with effector memory CD8^+^ T cells in both BPN and IPF datasets, underscoring the potential immunological relevance of these genes in disease modulation (Figure 8B,D).

### 2.8. GSEA Reveals Immune Activation in BPN and Fibrotic Remodeling in IPF Through Differential Enrichment of ANKRD23 and MME

**Figure 9:**
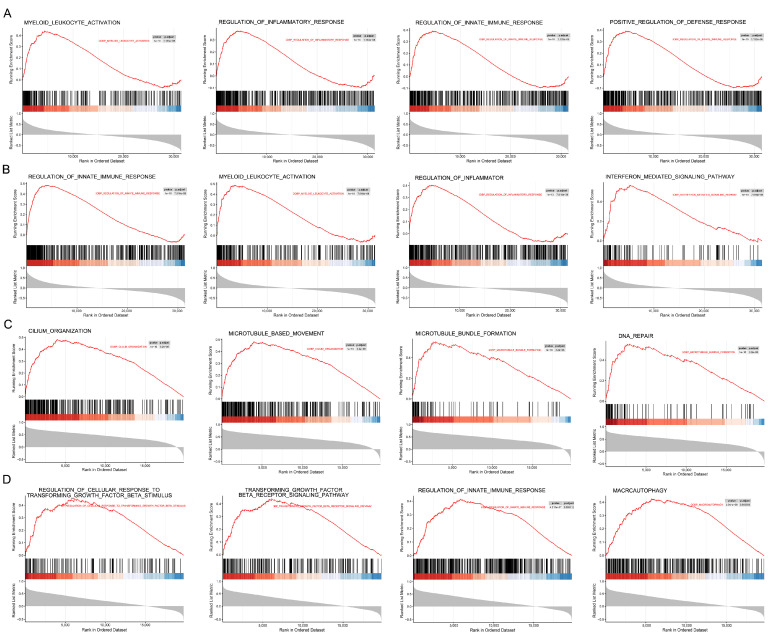
GSEA of *ANKRD23* and *MME* in BPN and IPF. (**A**): GSEA results for *ANKRD23* in the BPN; (**B**): GSEA results for *MME* in BPN; (**C**): GSEA results for *ANKRD23* in the IPF; (**D**): GSEA results for *MME* in IPF.

In the BPN cohort, GSEA revealed that *ANKRD23* was predominantly enriched in immune-related biological processes, including myeloid leukocyte activation, regulation of inflammatory response, regulation of innate immune response, and positive regulation of defense response (Figure 9A). Similarly, the *MME* gene in BPN was associated with pathways implicated in the regulation of innate immune response, myeloid leukocyte activation, regulation of inflammatory response, and the interferon-mediated signaling pathway (Figure 9B). In contrast, in IPF, *ANKRD23* showed enrichment in processes related to cellular structure and genome stability, such as cilium organization, microtubule-based movement, microtubule bundle formation, and DNA repair (Figure 9C). Meanwhile, the *MME* gene in IPF was enriched in pathways associated with regulation of cellular response to transforming growth factor-β (TGF-β) stimulus, the TGF-β receptor signaling pathway, regulation of innate immune response, and macroautophagy (Figure 9D).

### 2.9. External Data Set Verification and Experimental Verification

**Figure 10:**
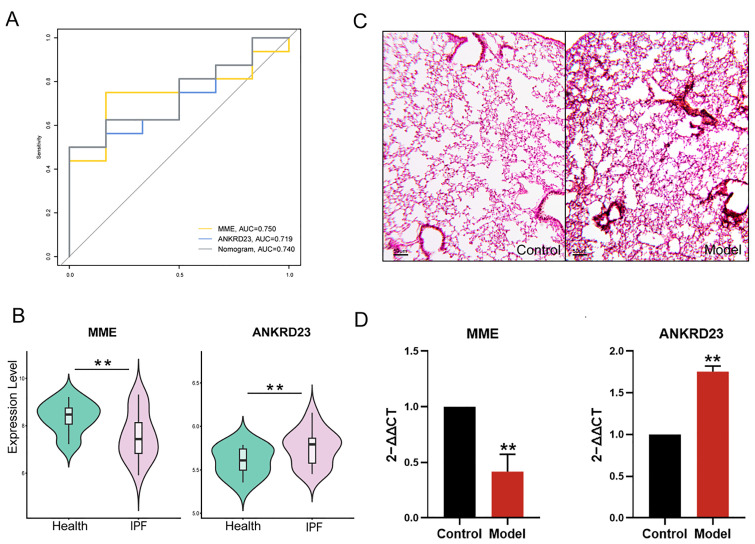
Evaluation and validation of the diagnostic value of *MME* and *ANKRD23* in IPF. (**A**): ROC curves of *MME* and *ANKRD23* for diagnosing IPF in the external dataset (GSE24206); (**B**): Violin plot of *MME* and *ANKRD23* expression in IPF patients and normal controls; (**C**): Representative HE-stained lung tissue sections from model and control groups; (**D**): qPCR validation of *MME* and *ANKRD23* expression levels in lung tissues. ** *p* < 0.01.

To evaluate the diagnostic potential of the identified key genes *MME* and *ANKRD23* in IPF, we performed receiver operating characteristic (ROC) curve analyses using an independent external dataset (GSE24206). The results demonstrated that both genes displayed favorable discriminatory performance, with areas under the curve (AUCs) of 0.740 and 0.719, respectively (Figure 10A). Consistent with the earlier predictive findings (Figure 7E), *MME* expression was significantly lower in patients than in controls, whereas *ANKRD23* exhibited markedly elevated expression levels (Figure 10B). Furthermore, quantitative real-time polymerase chain reaction (qPCR) validation revealed that *MME* was generally downregulated in lung tissues of the model group, while *ANKRD23* also showed reduced expression in lung tissues, corroborating the bioinformatics predictions (Figure 10C,D).

### 2.10. Identification of Potential Drug Interactors for ANKRD23 and MME

**Figure 11:**
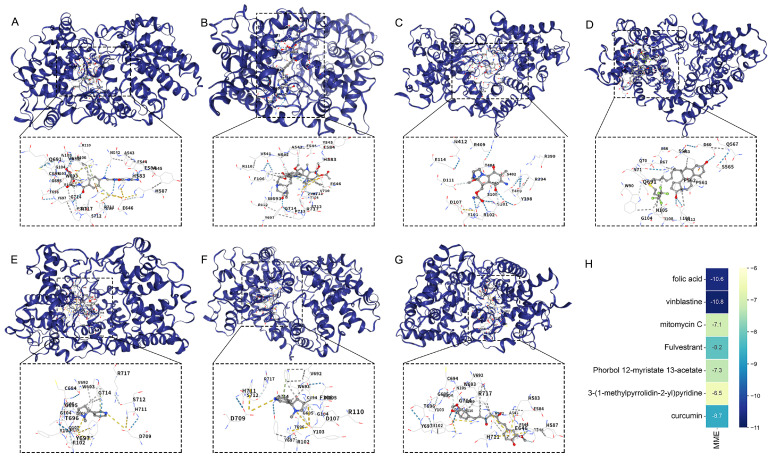
Predicted interactions between candidate compounds and targets. (**A**): Molecular docking result of folic acid with MME; (**B**): Molecular docking result of vinblastine with MME; (**C**): Molecular docking result of arbutin with MME; (**D**): Molecular docking result of mitomycin C with MME; (**E**): Molecular docking result of fulvestrant with MME; (**F**): Molecular docking result of phorbol 12-myristate 13-acetate with MME; (**G**): Molecular docking result of 3-(1-methylpyrrolidin-2-yl) pyridine with MME; (**H**): Heatmap of binding energies for all candidate compounds, illustrating their predicted affinities for MME.

In this study, the Enrichr was employed to predict potential drug interactions for the genes *ANKRD23* and *MME*. The analysis identified several candidate compounds that may interact with these genes, including folic acid, vinblastine, arbutin, mitomycin C, fulvestrant, phorbol 12-myristate 13-acetate, 3-(1-methylpyrrolidin-2-yl)pyridine, and curcumin. These compounds were highlighted based on their potential relevance to the biological pathways associated with *ANKRD23* and *MME* (Table 1). Molecular docking results indicated that, with the exception of 3-(1 methylpyrrolidin 2 yl) pyridine, which exhibited only moderate affinity for the receptor protein with binding free energies from −5.0 to −7.0 kilocalories per mole, all other compounds displayed strong binding interactions with binding free energies lower than −7.0 kilocalories per mole (Figure 11A–H).

## 3. Discussion

The central pathogenic mechanism of IPF involves repetitive alveolar epithelial injury coupled with dysregulated repair. Injured alveolar epithelial type II (AT2) cells activate fibroblasts through profibrotic mediators such as transforming growth factor-β (TGF-β) and recruit immune cells, particularly macrophages, via chemokine release, thereby initiating a self-amplifying cascade of epithelial injury, immune activation, and excessive extracellular matrix deposition that culminates in progressive collagen accumulation and irreversible lung remodeling [11,18]. Importantly, similar fibrotic programs are observed across multiple organs, including the liver and heart, where distinct initiating insults converge on shared pathological endpoints characterized by aberrant immune–stromal cell activation and collagen overproduction, reflecting conserved profibrotic pathways across tissues [9,19]. Current antifibrotic therapies, such as nintedanib and pirfenidone, primarily target downstream disease processes and slow functional decline without reversing established fibrosis or preventing disease initiation [12,13]. It is worth noting that the formation of BPN is often associated with persistent chronic low-grade inflammation, imbalance of local tissue repair response and tendency of interstitial fibrosis. Against this background, the present study focuses on biomarkers shared between BPN and IPF, aiming to identify upstream regulatory factors that may govern the transition from localized epithelial remodeling to diffuse fibrotic lung disease and serve as lung-specific molecular switches driving IPF initiation and progression.

The enrichment analysis of the co-DEGs of BPN and IPF showed that the co-DEGs shared between IPF and BPN are significantly enriched across multiple biological levels. Notably, these co-DEGs are clustered in myofibril and contractile fiber-related components, suggesting a shared propensity for cytoskeletal reorganization and aberrant extracellular matrix (ECM) remodeling in both conditions, processes that are central to fibrotic progression and tissue stiffness [9,15]. In parallel, significant enrichment was observed in pathways associated with viral infection. This finding raises the possibility that viral infections, or virus-induced immune memory responses, may act as common etiological triggers or modulators of the pathological microenvironment in both IPF and BPN. Increasing evidence supports a role for persistent or latent viral infections in driving chronic lung inflammation, epithelial injury, and fibroblast activation, thereby contributing to fibrotic remodeling [20,21,22]. Furthermore, the enrichment of enzyme activity-related terms, including lyase activity and metallopeptidase inhibitor activity, together with the significant involvement of the FoxO signaling pathway, highlights the potential importance of metabolic reprogramming and cellular stress-response mechanisms in the comorbid progression of these two diseases [23,24].

To identify more accurate biomarkers for BPN and IPF, we integrated differential gene expression analysis and WGCNA module analysis with LASSO, RF, and SVM learning algorithms. *ANKRD23* and *MME* were identified as potential hub biomarker genes shared by BPN and IPF. *MME*, also known as CD10 or neutral endopeptidase, is a key extracellular membrane-bound protease whose principal function is the degradation of a broad spectrum of bioactive peptides. Through this activity, *MME* plays a central role in the regulation of inflammatory responses, control of ECM turnover, and modulation of immune cell activation under both physiological and pathological conditions [25,26,27]. In the present study, we observed that the expression pattern of *MME* exhibits strikingly opposite trends in BPN and IPF, consistent with its context-dependent functional divergence. In patients with BPN, *MME* expression is significantly upregulated compared with healthy controls. As a benign lesion, elevated local *MME* expression in BPN may exert a protective regulatory function by hydrolyzing excessive pro-inflammatory mediators and chemotactic peptides—such as bradykinin and neurokinins—thereby fine-tuning the local inflammatory response. This controlled degradation facilitates appropriate immune cell activation and clearance, helps maintain a balanced immune microenvironment, and supports orderly tissue repair following injury [27,28]. In contrast, within the pathological context of IPF, *MME* activity is markedly reduced, resulting in impaired clearance of its substrates, including endothelin-1. Endothelin-1 is a potent pro-fibrotic and mitogenic peptide, and suppression of *MME* diminishes its degradative capacity, leading to abnormal accumulation within fibrotic lesions. In addition, *MME* is involved in the regulation of multiple protease systems that collectively maintain ECM homeostasis. Consequently, *MME* downregulation in IPF contributes to sustained activation of pro-fibrotic signaling pathways—such as non-canonical TGF-β–related circuits—and disrupts the balance between ECM synthesis and degradation. Together, these effects promote excessive ECM deposition and pathological tissue remodeling, thereby accelerating fibrotic progression [25,26]. Collectively, these findings suggest that *MME* plays divergent roles depending on the tissue repair context. In BPN, elevated *MME* expression may contribute to a regulated immune microenvironment conducive to effective repair, whereas in IPF, reduced *MME* expression may represent/constitute the loss of a critical regulatory constraint, thereby favoring impaired degradation of pro-fibrotic mediators, disrupted ECM homeostasis, and persistence of a fibrotic niche. This context-dependent behavior underscores the functional plasticity of *MME* and its potential relevance in shaping disease progression. However, the opposite expression patterns of *MME* in BPN and IPF indicate that it has limited standalone diagnostic value and must be interpreted in a disease-context-dependent manner rather than as an independent biomarker. In clinical practice, the utility of *MME* is grounded in its bidirectional expression pattern. On the one hand, when interpreted in conjunction with clinical and radiological findings, *MME* may serve as a supportive biomarker for distinguishing BPN—characterized by elevated expression during the inflammatory repair phase—from IPF, where expression is reduced during the fibrotic stage. On the other hand, incorporating *MME* into multi-biomarker panels or machine learning models enables the generation of robust probabilistic scores based on both the direction and magnitude of its expression, thereby mitigating the impact of biological heterogeneity. Ultimately, by quantifying the balance between tissue repair and fibrogenesis, *MME* enhances the discriminatory power of existing diagnostic strategies for patient stratification.

ANKRD23, also known as muscle ankyrin repeat protein-3 (MARP3 or DARP), is a member of the muscle ankyrin repeat protein (MARP) family. Proteins of this family are characterized by conserved ankyrin repeat domains and exhibit dual localization to the sarcomeric I-band and the nucleus, where they exert transcriptional regulatory functions [29]. ANKRD23 has been implicated in cellular stress responses, mechanotransduction, and muscle fiber regulation, acting as a potential mediator linking mechanical or metabolic stress to nuclear gene expression programs [30]. Although the role of *ANKRD23* in IPF has not been directly demonstrated, its molecular characteristics suggest a potential involvement in fibrosis-associated cell populations, such as myofibroblasts, which are highly responsive to mechanical cues and transcriptional reprogramming. In contrast, the other two members of the MARP family display clearer associations with tissue remodeling and fibrosis. ANKRD1 (CARP/MARP1) has been well established as a regulator of pathological cardiac remodeling and fibrosis, where it modulates stress-responsive transcriptional networks [29,31]. ANKRD2 (ARPP/MARP2) has been reported to participate indirectly in tissue remodeling processes, particularly through its roles in inflammation, oxidative stress, and skeletal muscle injury responses [32]. Compared with *ANKRD1* and *ANKRD2*, *ANKRD23* currently lacks direct experimental evidence linking it to fibrotic progression; however, given the shared structural features and partially overlapping functions within the MARP family, *ANKRD23* may possess latent or context-dependent profibrotic potential that warrants further investigation.

Having identified a set of key candidate genes that are dysregulated in both BPN and IPF, we then performed GSEA to investigate whether these shared molecular factors participate in common or distinct biological processes in each condition. *ANKRD23* and *MME* exhibited marked context-dependent functional divergence between the two conditions. In BPN, both genes were primarily enriched in immune-related processes, including myeloid leukocyte activation, regulation of innate and inflammatory immune responses, and positive regulation of defense responses, suggesting a role in maintaining a localized and potentially protective immune microenvironment [33]. In contrast, their functional profiles in IPF shifted toward pathological remodeling. *ANKRD23* was mainly associated with cytoskeletal organization and genome maintenance, such as cilium organization, microtubule-based movement, and DNA repair, whereas *MME* was enriched in fibrogenesis-related pathways, including TGF-β receptor signaling and macroautophagy. This functional divergence indicates that identical genetic factors may exert distinct biological effects depending on disease context—supporting controlled immune responses in BPN while contributing to aberrant tissue repair and fibrosis in IPF, consistent with the recognized links between fibrosis, dysregulated repair, and cancer-like biological behavior in IPF [9,15].

To facilitate clinical translation, this study employed Enrichr to predict drug–gene interactions for *ANKRD23* and *MME*, identifying candidate compounds including folic acid, vinblastine, arbutin, mitomycin C, fulvestrant, phorbol 12-myristate 13-acetate (PMA), 3-(1-methylpyrrolidin-2-yl)pyridine, and curcumin. Molecular docking analysis demonstrated that, with the exception of 3-(1-methylpyrrolidin-2-yl)pyridine, which exhibited only moderate affinity (−5.0 to −7.0 kcal/mol), all other compounds displayed strong binding potential to the target proteins (<−7.0 kcal/mol), suggesting their structural feasibility for interaction. Given that ANKRD23 is a nuclear transcription-related protein, its “druggability” may lie more in pathway or expression-level regulation rather than classical enzymatic inhibition. In contrast, MME (neprilysin/CD10), as a membrane-bound metalloendopeptidase, presents a clearer pharmacological target with established clinical strategy frameworks for similar enzymes. Among the candidates, curcumin and arbutin combine docking advantages with existing evidence in anti-inflammatory/anti-allergy research. Curcumin, for instance, exerts anti-inflammatory effects by inhibiting pathways such as NF-κB and downregulating multiple pro-inflammatory cytokines, and randomized double-blind studies suggest it may improve allergic rhinitis symptoms [34,35]. Arbutin has also demonstrated anti-inflammatory and anti-allergic activities in animal models of allergic rhinitis [36]. Furthermore, folic acid is implicated in immune homeostasis and the modulation of inflammatory markers, with systematic reviews indicating its potential influence on CRP, IL-6, and TNF-α levels. Notably, these flavonoids, and saponins are naturally derived bioactive compounds commonly found in traditional Chinese medicines, such as Jingfang Granules, and are generally associated with favorable safety profiles. In comparison [37], vinblastine and mitomycin C are cytotoxic anticancer agents, fulvestrant is an endocrine therapy for cancer, and PMA is a commonly used research tool for activating PKC/NF-κB pathways. Although these molecules may show strong docking signals, their feasibility for repurposing in chronic conditions like allergic inflammation is limited due to their specific indications and safety profiles [38,39].

Several limitations of the present study should be acknowledged. First, the etiologies of BPN and IPF are inherently heterogeneous and multifactorial, involving diverse environmental, genetic, and clinical determinants. As a result, a range of uncontrollable confounding factors may have influenced the observed molecular associations. Additionally, the use of different types of samples for analysis (peripheral blood for BPN and lung tissue for IPF) constitutes another limitation, as cross-tissue comparisons may limit mechanistic interpretation and introduce tissue-specific bias, potentially influencing the observed results due to inherent differences in biological context between blood and lung tissue. Second, owing to the limited availability of publicly accessible datasets, external validation of the identified genes was constrained by the lack of independent datasets specifically comprising benign pulmonary nodule samples. Third, due to the difficulty of obtaining human specimens, validation was performed in a mouse model; however, these results provide supportive rather than mechanistic evidence, and species differences may limit direct translational interpretation. Fourth, the experimental design was constrained by the absence of suitable animal models mimicking the self-limiting inflammatory lesions of BPN, limiting validation to the fibrotic phase (day 21 after bleomycin administration). Therefore, the current validation reflects an association with established fibrosis severity rather than early disease prediction or early biomarker potential. Finally, although multiple candidate drugs were predicted to interact with the identified targets, their therapeutic efficacy and translational potential remain speculative and require rigorous validation through subsequent preclinical experiments and well-designed clinical studies.

## 4. Materials and Methods

### 4.1. Data Sources

All data samples utilized in this study were sourced from the Gene Expression Omnibus (GEO) database (http://www.ncbi.nlm.nih.gov/geo (accessed on 1 October 2025)). From the GSE135304 dataset, a total of 17 patients with BPN were selected based on the following inclusion criterion: a maximum nodule diameter of ≥20 mm. In parallel, 17 healthy controls (HC) were randomly sampled from the same dataset to serve as the control group. The experimental cohort for IPF (GSE10667) consists of lung tissue samples from 31 IPF patients and 15 HC. For the validation cohort, lung tissue samples were obtained from the GEO dataset GSE24206, which includes 16 IPF patients and 6 HC. All datasets are publicly available and accessible through the GEO database.

### 4.2. Identification of Differentially Expressed Genes

Differentially expressed genes (DEGs) between the relevant groups (BPN vs. HC, IPF vs. HC) were identified using the R package limma (version 4.4.3) within the Bioconductor framework. Raw gene expression data from the GEO datasets were preprocessed by performing background correction, normalization, and quality control steps [40]. The expression levels were log-transformed to stabilize variance and improve comparability across samples. Differential expression analysis was conducted by fitting a linear model to the data, followed by empirical Bayes moderation to estimate gene expression differences. In the BPN group, genes with |log2 fold change| > 0.263 and an FDR-adjusted *p*-value < 0.05 were considered significantly differentially expressed, while in the IPF group, genes with |log2 fold change| > 0.5 and an FDR-adjusted *p*-value < 0.05 were identified as DEGs. Volcano plots were generated to visualize DEGs.

### 4.3. Protein–Protein Interaction Network

The protein–protein interaction (PPI) network of common DEGs between BPN and IPF was constructed using the STRING database (https://string-db.org/ (accessed on 15 October 2025)) and visualized with Cytoscape (version 3.9.1). Interactions with a confidence score > 0.4 were included, and genes were ranked based on their DEGREE value.

### 4.4. Enrichment Analysis of Common Differentially Expressed Genes

Enrichment analysis of the common DEGs between BPN and IPF groups was performed using KEGG pathway and Gene Ontology (GO) functional annotation via the online platform Bioinformatics.com.cn (https://www.bioinformatics.com.cn/ (accessed on 15 October 2025)). Pathways and GO terms with adjusted *p*-values < 0.05 were considered statistically significant. The results were visualized through bar plots, dot plots, and network diagrams to identify key biological pathways and processes shared between the two diseases.

### 4.5. Weighted Gene Co-Expression Network Analysis (WGCNA)

WGCNA was conducted using R (version 4.4.3) to identify co-expression modules associated with BPN and IPF. Genes with low variance were filtered out, and only the top 25% most variable genes were retained for network construction. The soft-thresholding power was set to 0.8 to ensure a scale-free network. An adjacency matrix was constructed based on pairwise Pearson correlation coefficients, and this matrix was transformed into a topological overlap matrix (TOM) to assess gene interconnectedness. Gene clustering was performed using dynamic tree cutting, with a minimum module size of 75 genes. Modules were then correlated with clinical traits to identify those most relevant to BPN and IPF. The genes included in the modules with the strongest correlation coefficients for both diseases were intersected with the DEGs identified earlier. A Venn diagram was then generated to visualize the overlap between these gene sets. The visualization was performed using the Wei Sheng Xin platform (https://www.bioinformatics.com.cn (accessed on 15 October 2025)), a bioinformatics tool for generating interactive Venn diagrams.

### 4.6. Machine Learning to Identify Target Hub Genes

Three machine learning algorithms were employed: the Least Absolute Shrinkage and Selection Operator (LASSO) regression model, Random Forest (RF), and Support Vector Machine (SVM), to identify genes with the highest diagnostic value. The results were subsequently visualized using a nomogram. The final set of genes was derived by taking the intersection of the top-ranked genes identified by all three models.

### 4.7. Immune Infiltration Analysis

The immune cell infiltration in the gene expression profiles associated with BPN and IPF was assessed using ssGSEA. First, the GSVA package (version 1.50.5) was used to calculate the enrichment scores for various immune cells. Subsequently, vioplot was employed to generate violin plots, comparing the abundance and proportion of infiltrating immune cells between the two groups. To determine the statistical significance of differences in the proportions of 28 distinct immune cell types between the experimental and control groups, a Student’s *t*-test was performed, with a *p*-value < 0.05 considered statistically significant.

### 4.8. Single-Gene GSEA

This study used single-gene gene set enrichment analysis (GSEA) to investigate the functional roles of the identified biomarkers in both disease conditions and to explore their potential downstream targets. Samples were categorized into high- and low-expression groups based on the median expression levels of the two genes of interest. The analysis utilized the “c2.cp.kegg_legacy.v2025.1.Hs.symbols.gmt” gene set database as the reference.

### 4.9. Construction and Validation of a Nomogram Model with ROC Curve Analysis for Diagnostic Biomarkers

A nomogram was constructed using the rms package in R (version 4.4.3), incorporating the selected biomarkers. In the model, the “Score” represents the individual contribution of each candidate gene, while the “Total Score” is the cumulative sum of all gene scores. The nomogram’s predictive accuracy was assessed through calibration curves. To further evaluate the diagnostic performance of the biomarkers, Receiver Operating Characteristic (ROC) curves were generated using the pROC package (version 1.18.5), and the area under the curve (AUC) was calculated. A value of AUC > 0.7 was considered indicative of substantial diagnostic value.

### 4.10. Bleomycin-Induced IPF Model in Mice

IPF was induced in C57BL male mice by administering bleomycin (BLM; MedChemExpress (Monmouth Junction, NJ, USA), Cat# HY-108345) via intratracheal instillation. Mice were anesthetized using isoflurane (MedChemExpress, Cat# HY-A0134) and placed on a heating pad to maintain body temperature. A single dose of bleomycin (5 mg/kg body weight) was instilled into the trachea of each mouse, and control mice received an equivalent volume of sterile saline. Mice were monitored daily for signs of distress, and their body weight was recorded throughout the study period. After 21 days, the mice were euthanized, and lung tissues were collected for histological analysis to assess the extent of fibrosis. 

### 4.11. qPCR

Total RNA was extracted from murine lung samples using an RNA purification kit and quantified. Complementary DNA (cDNA) was synthesized through reverse transcription of the RNA. Quantitative PCR (qPCR) was conducted, and data were analyzed using the 2^−ΔΔCt^ method. The primer sequences used are listed in Table S1.

### 4.12. Gene–Drug Prediction

**Figure 12:**
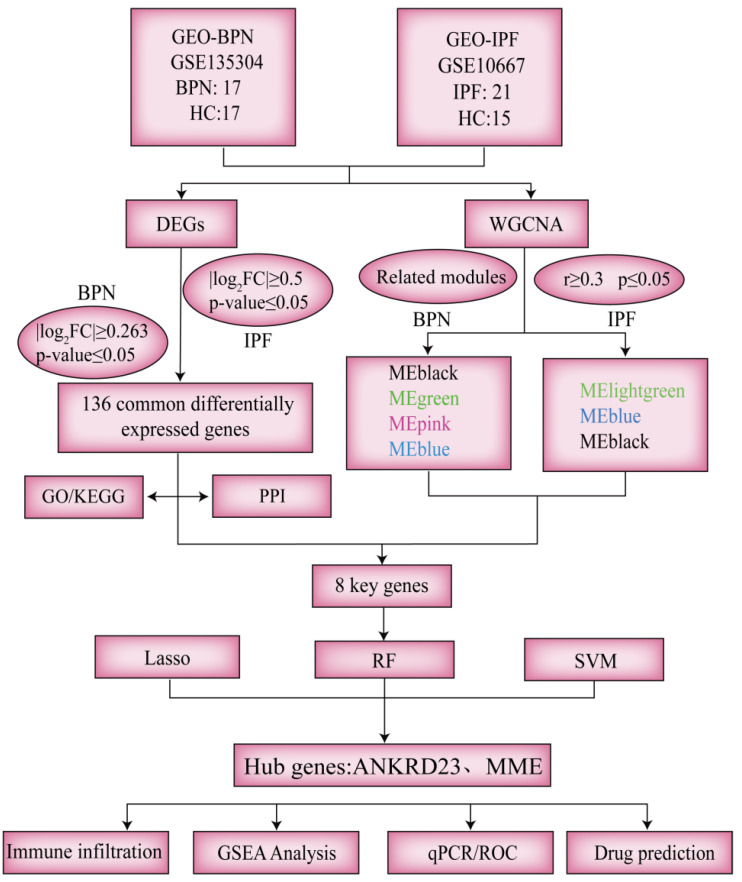
Technical Roadmap for Experimental Design.

Potential therapeutic agents targeting the candidate genes were predicted using the DSigDB module of the Enrichr platform (https://maayanlab.cloud/Enrichr/ (accessed on 26 October 2025)). Compounds with *p*-values < 0.05 were retained as statistically significant candidates [41,42]. Molecular docking was employed to analyze the intermolecular interactions between the predicted therapeutic compounds and the target proteins encoded by the core genes identified in this study. Small-molecule ligand data were obtained from the PubChem database (http://pubchem.ncbi.nlm.nih.gov (accessed on 26 October 2025)), and the three-dimensional structures of macromolecular receptors were retrieved from the Protein Data Bank (PDB; http://www.rcsb.org (accessed on 26 October 2025)). Docking simulations were performed using CB-DOCK2 (https://cadd.labshare.cn/cb-dock2/index.php (accessed on 26 October 2025)) to predict binding energies and potential interaction sites. Subsequently, ligand–receptor binding modes were visualized to elucidate key molecular interactions. The overall workflow of this study is illustrated in Figure 12.

## 5. Conclusions

This work ultimately identified *MME* and *ANKRD23* as potential diagnostic biomarkers linking BPN and IPF. The study further highlighted their interactions across multiple cellular processes, including inflammation, interstitial secretion, and immune regulation. These findings provide important insights for the early clinical diagnosis of BPN-to-IPF progression and for the development of interventional therapeutics. In the future, through in-depth investigation of the functions and mechanisms of these genes combined with experimental validation, we anticipate the formulation of precise therapeutic strategies and the provision of a scientific basis for developing preventive strategies targeting high-risk populations, thereby delaying or even preventing the onset and progression of IPF.

## Figures and Tables

**Table 1:** Drug prediction based on Enrichr website for *ANKRD23* and *MME*.

Drugs	Overlap	*p*-Value	Combined Score	Targets
folic acid	1/194	0.02	405.06	*MME*
vinblastine	1/227	0.02	331.66	*MME*
arbutin	1/282	0.03	250.88	*ANKRD23*
mitomycin C	1/308	0.03	223.71	*MME*
Fulvestrant	1/310	0.03	221.83	*MME*
Phorbol 12-myristate 13-acetate	1/483	0.05	123.18	*MME*
3-(1-methylpyrrolidin-2-yl)pyridine	1/499	0.05	117.87	*MME*
curcumin	1/528	0.05	109.15	*MME*

## Data Availability

The datasets presented in this study can be found in online repositories. The names of the repository/repositories and accession number(s) can be found below: https://www.ncbi.nlm.nih.gov/geo/ (accessed on 1 October 2025), GSE135304, GSE10667, GSE24206.

## Supplementary Materials

The following supporting information can be downloaded at https://www.mdpi.com/article/10.3390/ijms27083647/s1.
