# Supplementary material for: From Local Tissue Repair to Fibrosis: Deciphering Gene Co-Expression Networks in Benign Pulmonary Nodules and Idiopathic Pulmonary Fibrosis Comorbidity via Bioinformatics and Machine Learning
Source: Int J Mol Sci. 2026 Apr 19;27(8):3647. doi: 10.3390/ijms27083647 (PMC13116825; doi:10.3390/ijms27083647)
Supplement: Supplementary file 1 [file ijms-27-03647-s001.zip › Table S1.pdf]

Table S1. Real-time quantitative PCR primer sequences.

Symbol	Primer Sequence
MME	forward: 5'-GAACCTCCCGGGACTCTGG-3'
	reverse: 5'-CACATCCCGACCAATGAGCG-3'
ANKRD23	forward: 5'-CACAGCGGAGTGAAAAGCAT-3'
	Reverse: 5'-AGCCTCCAGGATCAGGAACT-3'
