# Supplementary figures and images for: From Local Tissue Repair to Fibrosis: Deciphering Gene Co-Expression Networks in Benign Pulmonary Nodules and Idiopathic Pulmonary Fibrosis Comorbidity via Bioinformatics and Machine Learning
Source: Int J Mol Sci. 2026 Apr 19;27(8):3647. doi: 10.3390/ijms27083647 (PMC13116825; doi:10.3390/ijms27083647)

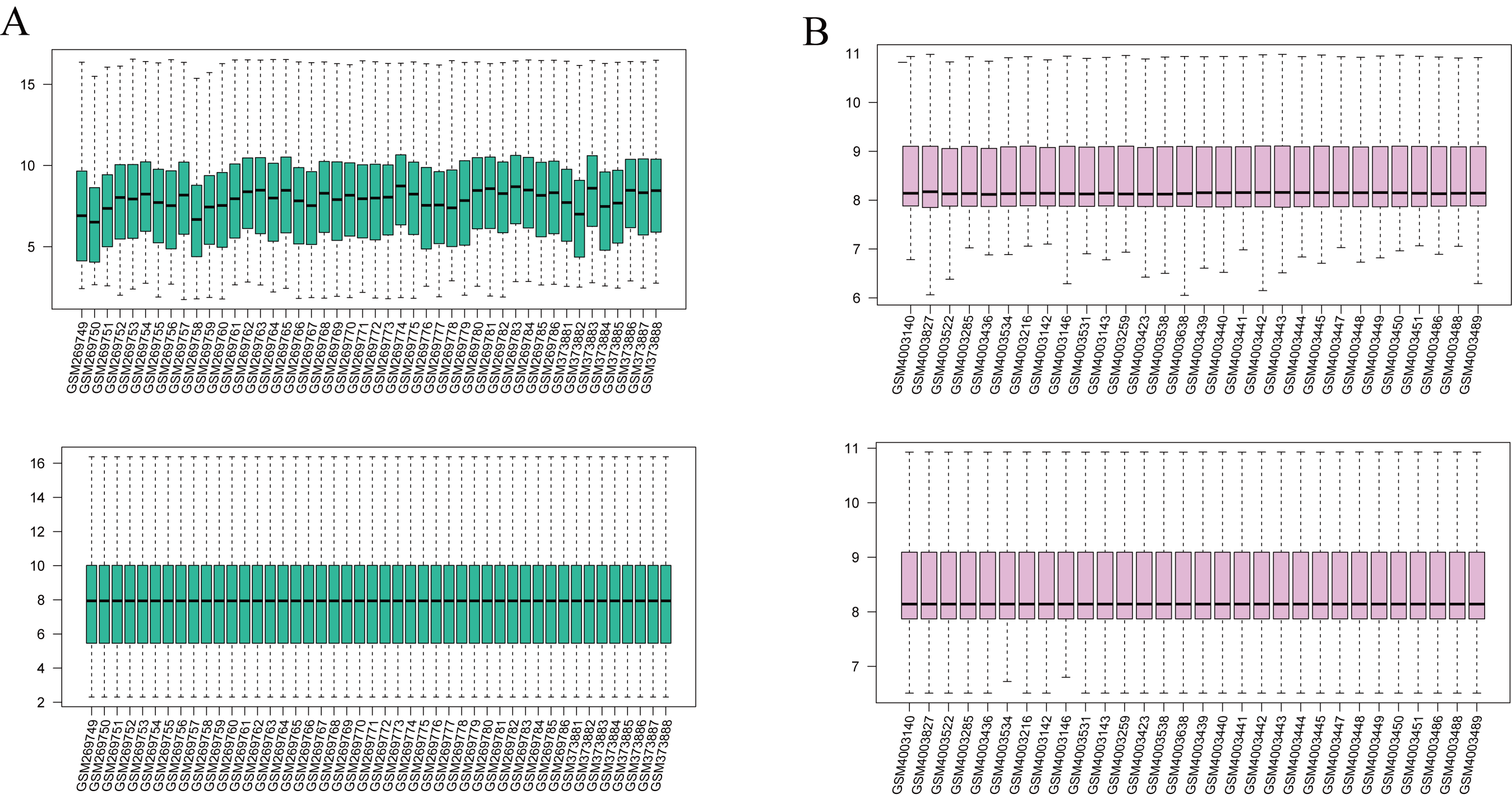

Supplement: Supplementary file 1 [file ijms-27-03647-s001.zip › Figure S1.tiff]
